# Non-significant association between − 330 T/G polymorphism in interleukin-2 *gene* and chronic periodontitis: findings from a meta-analysis

**DOI:** 10.1186/s12903-020-1034-8

**Published:** 2020-02-19

**Authors:** Felipe Rodolfo Pereira da Silva, Juliana Gomes Galeno, Alessandro Luiz Araújo Bentes Leal, Reyce Santos Koga, Nayana Yared Batista, Silvânia da Conceição Furtado, Daniel Fernando Pereira Vasconcelos, Marcelo Diniz Carvalho, José Fernando Marques Barcellos

**Affiliations:** 1 Doctorate Post-Graduation Program in Basic and Applied Immunology, Federal University of Amazonas, Manaus, Amazonas Brazil; 2 Department of Morphology, Federal University of Amazonas, General Rodrigo Octavio Jordão Ramos Avenue, 1200, Coroado, Manaus, Amazonas CEP: 69067-005 Brazil; 3 Laboratory of Histological Analysis and Preparation (LAPHIS), Federal University of Piaui, Parnaiba, PI Brazil; 4 Department of Periodontology, Amazonas State University, Manaus, Amazonas Brazil

**Keywords:** Periodontal disease, Cytokine, Odds ratio, Risk factor, Allele

## Abstract

**Background:**

Chronic periodontitis (CP) is an immune-inflammatory disease that promotes tissue damage around the teeth. Among the several inflammatory mediators that orchestrate the periodontitis, there is the interleukin (IL)-2. Genetic variations in IL2 *gene* may be associated with the risk and severity of the disease. Contrary results are available in the literature with inconclusive findings and none meta-analysis to gather these data.

**Methods:**

A literature search was performed for studies published before June 11, 2019 in diverse scientific and educational databases. The data was extracted by two investigators and the statistical evaluation was performed by Review Manager statistical program with heterogeneity (I^2^) and Odds Ratio (OR) with 95% of Confidence Intervals (CI) calculations and a sensitive analysis to assess the accuracy of the obtained results. The publication bias was evaluated by Begg’ and Egger’s test with Comprehensive meta-analysis software. The value of *P* < 0.05 was considered as significant.

**Results:**

Five studies were identified in diverse ethnical groups with 1425 participants. The − 330 T/G polymorphism in IL2 *gene* was not significantly associated with CP in allelic evaluation (*P* > 0.05) as well as in the genotypic comparisons (*P* = 0.15). The Begg’s test and the linear regression Egger’s test did not show any evidence of publication bias risk (*P* > 0.05) which was corroborated by the absence of obvious asymmetry in Funnel plot graphic.

**Conclusions:**

This meta-analysis showed a non-significant association between − 330 T/G polymorphism in IL2 *gene* and CP in any allelic evaluation.

## Background

Periodontitis is characterized as a clinical condition caused by accumulative dental plaque in periodontium and host-immune response with tissue damage resulting in possible teeth loss [1]. The disease reached significant high worldwide prevalence which 10% of the global population has been affected by the severe form of the disease [2]. Moreover, in Norway, 49.5% from 1911 patients had periodontitis [3] which the disease has affected 64.7 million of people into the United States between 2009 and 2012 [4] and had elevated prevalence in Italy [5] and in Brazilians [6].

The disease receives distinct classifications, which the chronic and aggressive periodontitis forms (CP and AgP, respectively) are the most common in clinical with the CP characterized by slow and swift progression reaching subjects with increased mean of age [7].

Several factors are involved in periodontitis development and progression from poor oral hygiene [8] to specific bacterial species in subgingival region [9] or even genetic factors in inflammatory mediators [10, 11]. Into the diverse molecules involved in host-immune response during infections or inflammation there is the interleukin (IL)-2. This cytokine is involved in both induction as well as termination of immune response [12] which IL-2 may promote the suppression of inflammation due to the T cells regulation [13]. Indeed, the therapy by low-dose IL-2 or the recombinant form of IL-2 was observed as an effective approach to autoimmune conditions and inflammatory diseases [14] as well as the suppression of tumor growth in pancreatic cancer [15].

Taken the IL-2 and periodontitis, there are contradictory findings available in the literature that regarding the association between increased IL-2 levels and the disease. Some authors have showed significant higher levels of this cytokine in gingival fluid from patient with periodontitis than healthy controls [16]. However, others authors have found contrary results [17].

These contradictory findings also have been observed in genetic evaluations. IL2 *gene* is located in the chromosome 4q26-q27 region which polymorphisms in this *gene* were first described by Jonh et al. [18] represented by two single-nucleotide polymorphisms at − 330 and − 384 promoter regions, consequently affecting the IL-2 expression. The first study focusing in genetic variations into the IL2 *gene* and periodontitis was published in 2002 by Scarel-Caminaga et al. [19] and the own study have presented divergent findings. Firstly, the authors found a non-significant association between the − 330 T/G polymorphism and periodontitis (*P* > 0.05), the significant relationship only was observed when the control group was combined with moderate periodontitis group. Then, the polymorphism was associated with the severe form of the disease. Likewise, Li et al. [20] identified significant association between this genetic variation and periodontitis in both allelic and genotypic analyses. However, after logistic regression test, the polymorphisms was not associated with periodontitis.

Contradictory findings in genetic studies may represent a challenge to research and seen the lack of a study that gather those findings, this study aimed to perform a meta-analysis approaching the results on the − 330 T/G polymorphism in IL2 *gene* and CP.

## Methods

To perform this current meta-analysis the recommended PRISMA (Preferred Reporting Items for Systematic Reviews and Meta-Analyses) statement was followed [21].

### Eligibility criteria

To be included in the current meta-analysis, the articles should bring studies which met all the following criteria: (1) Evaluation of − 330 T/G polymorphism in IL2 *gene* with periodontitis in humans; (2) Studies performed by case/control design; (3) The case patients have received diagnosis of CP confirmed by clinical manifestations or radiographic findings as previously described [22] and control patients had healthy periodontal clinical evaluation; (4) Genotypic frequency has been documented; (5) The participants included into the allelic and genotypic evaluations did not present pregnancy or systemic disorders (diabetes or auto-immunity disease).

### Search strategy

Two investigators independently have retrieved the available literature for studies that analyzed any possible association between the − 330 T/G in IL2 *gene* with periodontitis in human beings. The databases used in the systematic search were the following: China DATABASE, Google Scholar, PubMed and Web of Science. The authors used a combination of keywords or Medical Subject Headings (MeSH) as following: [(interleukin or cytokine or interleukin-2 or IL-2) and (genetic variation or rs2069762 polymorphism or − 330 T/G polymorphism) and (periodontitis or periodontal disease or chronic periodontitis)]. No language restriction have been used in the systematic search that have approached studies published before June 11, 2019. The abstracts of the screened studies and their references were revised by the investigators to identify some potential additional studies.

### Data collection

Two authors independently reviewed all the identified studies in the systematic search and have extracted the data by use of a standardized form that composed the table of characteristics of included studies. In attempt to assess the methodological quality of the included studies, the guidelines for systematic reviews of periodontal genetic association studies proposed by Nibali [23] have been used, studies that obtained less than 10 scores were excluded.

### Statistical analysis

The Review Manager software version 5.3 (RevMan, Nordic Cochrane Centre, The Cochrane Collaboration, 2012) for systematic reviews and meta-analyses was the statistical program used in the calculations. On the other hand, the publication bias have been evaluated by the Comprehensive Meta-analysis statistical software version 3.3.070 (2014), available on-line as a trial.

The presence or absence of true heterogeneity (I^2^), calculated by the chi-squared Q-based statistical test and by analysis of the Funnel plot graphic for heterogeneity. When the observed value of I^2^ presented a non-statistical significance (I^2^ < 50%, *P* > 0.05) the authors used the Fixed-effect model for the pooled Odds Ratio (OR) calculation. When I^2^ presented a statistical significant value (I^2^ > 50%, *P* < 0.05) the Random-effects statistical model was used for the OR calculations. The *P* value < 0.05 was considered as significant. To determinate the exact influence of the genetic variation in the meta-analysis calculations, six genetic models evaluated taking in base “M” as a mutant allele and “m” allele as a wild-type allele were calculated. Allelic comparisons: (I) M versus m -, (II) m versus M; Genotypic comparisons: (III) MM versus mm, (IV) mm versus MM; and the combinations among the genotypic variations: (V) MM versus mm + Mm and (VI) Mm versus MM + mm. The Begg’s test and Egger’s linear regression test (with *P* < 0.05) were the statistical analyses used to estimate the potential publication bias in this meta-analysis, the Funnel plot asymmetry was also considered. In addition, the authors have performed a sensitivity analysis to verify the robustness of the pooled results. There was the omission of one included study at a time to detect individual effects on the overall analyses. All the included data in the studies have been dichotomous data expressed as OR with 95% of confidence intervals (CI) to determinate the possible association between the polymorphism in IL2 *gene* and periodontitis.

## Results

### Characteristic of included studies

**Fig. 1:**
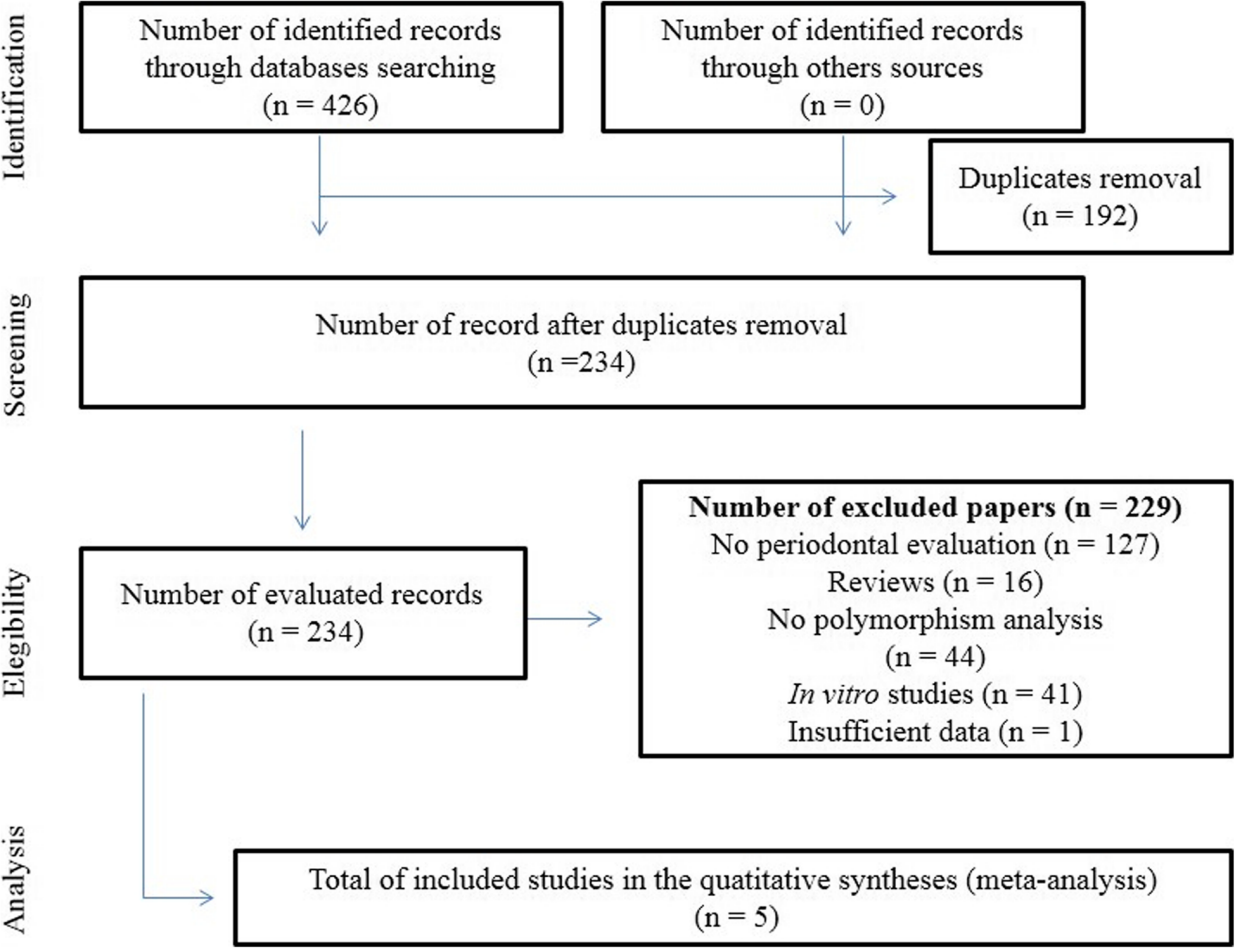
Flow diagram for identification, screening, selection and inclusion of the studies in this meta-analysis

At the finish of the systematic search, five articles [19, 20, 24–26], published between 2002 and 2019 met the inclusion criteria and therefore have been included in the meta-analysis (Fig. 1). The studies have been performed in three different ethnic groups: Caucasian [25, 26], Asian [20, 24] and Mixed population [20]. One article [20] have subdivided the case patients in two forms of chronic periodontitis: moderate (I) and severe (II), more details are available in Table 1. Seen this data, this current meta-analysis is composed by diverse studies in 1425 participants (505 case patients and 920 healthy controls). The PRISMA checklist with all the steps of this meta-analysis is available in Additional file 1: Table S1.

**Table 1:** Characteristics of included studies in this current meta-analysis

First Author and Reference	Year of Publication	Country	Ethnicity	Sample Size (Cc/Co)
Majumder [24]	2019	India	Asian	157/200
Li [20]	2012	China	Asian	122/532
Reichert [25]	2009	Germany	Caucasian	*58/69*
Scarel-Caminaga [19]	2002	Brazil	Mixed	69/44
Vahabi [26]	2017	Iran	Caucasian	99/75
First Author and Reference	Year of Publication	Genotypic Frequency (Cc- GG,TG,TT/Co- GG,TG,TT)	Age (Cc/Co)	Score and HWE respect (*X*^2^, P*value*)
Majumder [24]	2019	72,62,23/90,79,31	41.59 ± 11.12/38.41 ± 9.48	14/Yes (X^2^ < 3.84, *P* > 0.05)
Li [20]	2012	40,14,68/43,111,378	38.00–69.00	14/No (X^2^ > 3.84, *P* < 0.05)
Reichert [25]	2009	8,15,35/16,51,64	49.1 ± 9.6/46.7 ± 10.7	14/No (NI)
Scarel-Caminaga [19]	2002	4,29,36/2,16,26	36.9 ± 11.2(I)-43.6 ± 14.4(II)/43.2 ± 14.0	14/NI
Vahabi [26]	2017	0,0,99/0,0,75	40.28 ± 12.73/31.67 ± 12.00	12/NI

*Cc* Case patients, *Co* Healthy Controls, *I and II* Both studies performed by Scarel-Caminaga [18], *HWE* Hardy Weinberg Equilibrium with based on X^2^ test value and P*value* > 0.05, *HWE* Evaluated in case and control groups combined, *NI* Not informed

### Meta-analysis

**Fig. 2:**
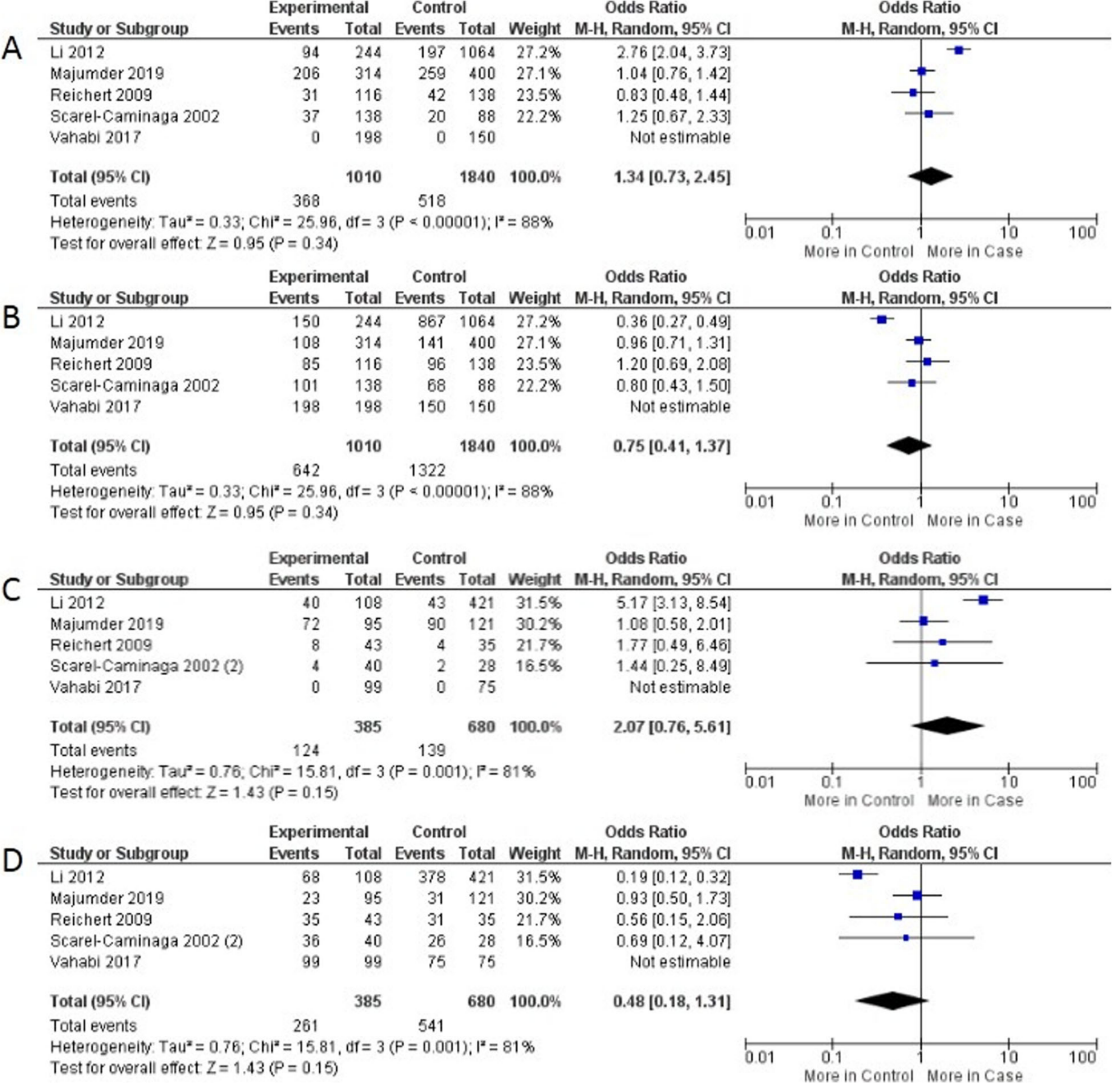
Forest plot of comparison between the mutant allele versus the wild type allele (**a**), the wild type allele versus mutant allele (**b**), the homozygous mutant genotype versus the homozygous wild type genotype (**c**) and the homozygous wild type genotype versus the homozygous mutant genotype (**d**) in the -330 T/G polymorphism and CP risk

The meta-analysis calculation showed a non-significant association between the − 330 T/G polymorphism in IL2 *gene* and CP in any allelic evaluation (Fig. 2). Likewise, non-significant results have been obtained in genotypic calculations which neither the mutant homozygous genotype was not associated with the disease risk (OR = 2.07, 95% CI: 0.76, 5.61, *P* = 0.15) nor wild type homozygous genotype was not associated with controls (OR = 0.48, 95% CI: 0.18, 1.39, P = 0.15). All calculations was performed by means of the Random-effects statistical model due to increased I^2^ (Table 2) but not for Caucasian group evaluation, which the calculations have been obtained by Fixed-effect statistical model. Moreover, a stratified analysis based in ethnicity were performed as showed in Table 2.

**Table 2:** Meta-analysis of comparison to − 330 T/G polymorphism in Interleukin-2 gene and chronic periodontitis risk (allelic and genotypic comparisons)

Comparison (n)	OR (95% CI)	P*value* (z test)	I^2^	P*heterogeneity*	Statistical model used
Overall (*n* = 5)					
M versus m	1.34 (0.73, 2.45)	0.34	88%	*P* < 0.00001	R
m versus M	0.75 (0.41, 1.37)	0.34	88%	*P* < 0.00001	R
MM versus mm	2.07 (0.76, 5.61)	0.15	81%	0.001	R
mm versus MM	0.48 (0.18, 1.31)	0.15	81%	0.001	R
MM versus Mm + mm	2.59 (0.62, 10.85)	0.19	93%	*P* < 0.00001	R
Mm versus MM + mm	0.70 (0.41, 1.20)	0.19	67%	0.03	R
Caucasian (*n* = 2)					
M versus m	0.83 (0.48, 1.44)	0.52	NA	–	F
m versus M	1.20 (0.69, 2.08)	0.52	NA	–	F
MM versus mm	1.77 (0.49, 6.46)	0.39	NA	–	F
mm versus MM	0.56 (0.15, 2.06)	0.39	NA	–	F
MM versus Mm + mm	2.90 (0.82, 10.27)	0.10	NA	–	F
Mm versus MM + mm	0.36 (0.17, 0.76)	0.008	NA	–	F
Asian (*n* = 2)					
M versus m	1.69 (0.65, 4.42)	0.28	95%	*P* < 0.00001	R
m versus M	0.59 (0.23, 1.54)	0.28	95%	*P* < 0.00001	R
MM versus mm	2.58 (1.74, 3.81)	*P* < 0.00001	93%	0.0001	R
mm versus MM	0.39 (0.26, 0.57)	*P* < 0.00001	93%	0.0001	R
MM versus Mm + mm	3.18 (0.34, 29.37)	0.31	98%	*P* < 0.00001	R
Mm versus MM + mm	0.72 (0.36, 1.45)	0.36	72%	0.06	R

*OR* Odds Ratio, *I*^*2*^ Heterogeneity, *M* mutant allele, *m* wild type allele, *R* Random-effects statistical model, *F* Fixed-effect statistical model, *NA* Not applicable by limited statistical power

### Sensitive analysis and publication bias

**Fig. 3:**
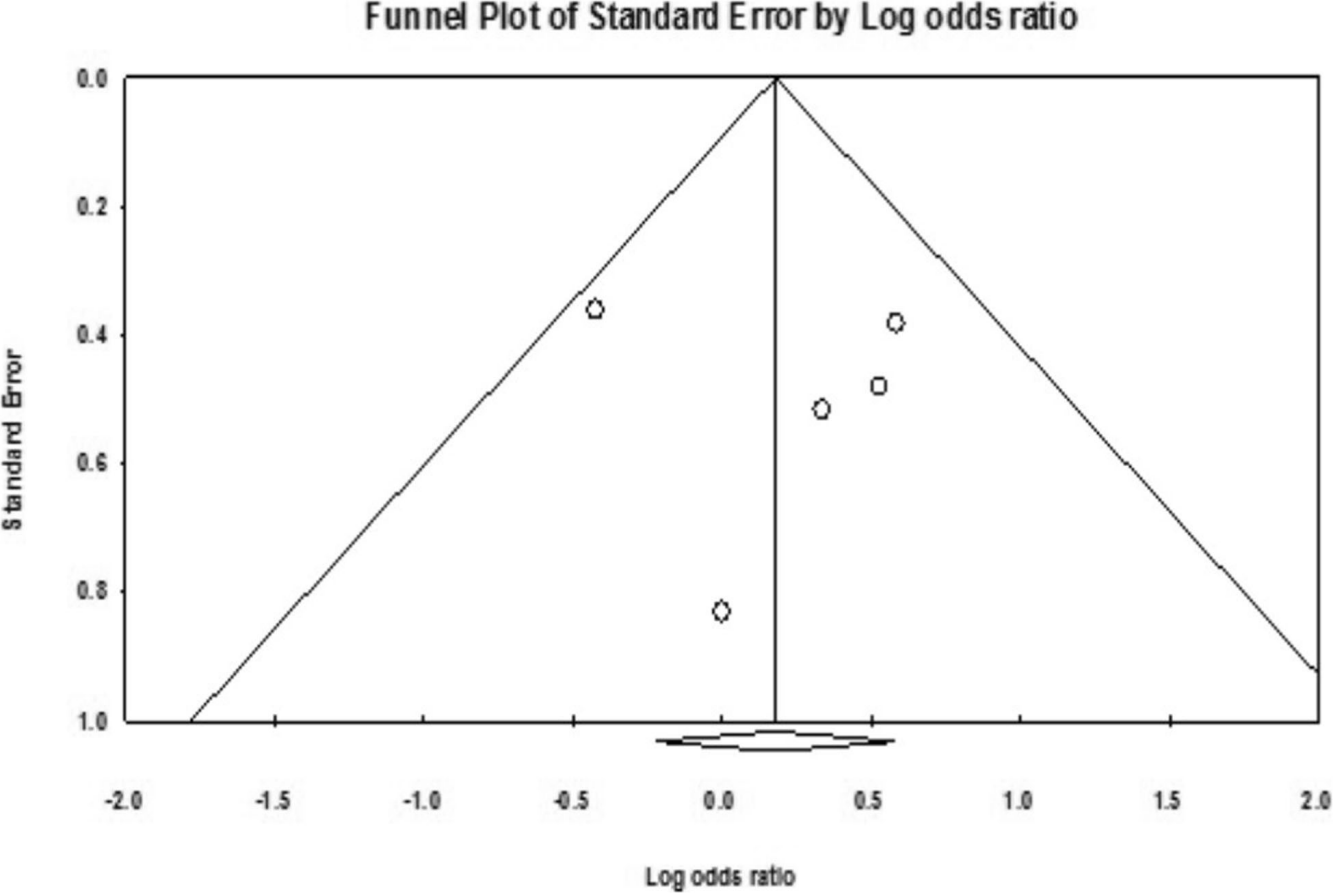
Funnel plot for publication bias in this current meta-analysis

The individual effect of included studies was estimate by a sensitivity analysis. Each study was omitting at the time to assess the possible impact on pooled OR value. No single study has changed the pooled OR value, quantitatively. It has suggested that the results from this current meta-analysis are accurate. No publication bias was found in this meta-analysis which is demonstrated by Begg’s test and Egger’s linear regression test in the allelic evaluation on the − 330 T/G polymorphism in IL2 *gene* and CP (*P* = 0.69 and *P* = 0.43, respectively). In addition, there was no asymmetry in the funnel plot for publication bias validating the tests performed (Fig. 3).

## Discussion

This is the first meta-analysis to approach the association between the aforementioned polymorphism and periodontitis. Periodontitis is a high prevalent oral disease that may carry out teeth loss. Studies in twins demonstrated the genetic role in periodontitis development [27], which the risk of the disease is increased when influenced by genetic polymorphism in host-immune molecules. Several previous meta-analyses bring results on the possible association among polymorphisms in IL1A [28, 29], IL1B [30, 31], IL6 [32], IL10 [33] and IL17A/F [34] *genes* and diverse clinical aspects of periodontitis, as well as dental implant failure [35].

Meta-analysis is considered as a statistical tool in genetic studies because this type of research may containing what is classify as small effects or limited coverage of genetic variability. Hence, the use of meta-analyses have been increased by potent capacity to detect significant associations among studies, mainly in meta-analyses with larger sample size [36].

The presented meta-analysis showed a non-significant association between this genetic variation and the disease in any allelic evaluation. This finding is according with a previous study, which the − 330 T/G polymorphism in the aforementioned IL was not associated with early dental implant failure [37] or type 1 diabetes [38]. Genetic variations in IL2 may contribute with inflammatory processes as seen in results which the amount of IL-2 protein levels were over three times for homozygous individuals for G allele in − 330 T/G polymorphism than T/T and T/G individuals in a CD3/CD28-stimulated peripheral blood lymphocytes [39].

IL-2 plays important role during inflammation by the increasing Natural Killer cytolytic activity and the differentiation of regulatory T cells [40]. The persistent IL-2 stimulation induces effectiveness in the expansion of memory T cells cytotoxic development [41] and appears to promote the activation-induced death cell of lymphocytes [42]. These evidences taken together may highlight the real role of IL-2 during periodontitis.

In addition, we consider that racial differences and ethnicity may influence the role of genetic variations in cytokines *genes* [43].

This meta-analysis has attempted to evaluate the influence of different ethnic groups in the results. As showed by Table 1, the included studies were performed in different ethnical groups which Mixed population was represented by 1 single study; and Caucasian and Asian ethnicities have been represented by two studies (*n* = 2). Therefore, we have performed calculations for a stratified analysis based in these both ethnical groups.

There was a major prevalence of T allele in − 330 T/G polymorphism in white individuals from United States population in comparison with African-American individuals [44] and presented the Minor Allele Frequency (MAF) = 0.0656 in American and African Ancestry [45]. In individuals from East Asian, the MAF of T allele was 0.6452, higher than American. The meta-analysis calculations showed a non-significant association between the − 330 T/G polymorphism in IL2 *gene* and periodontitis in the ethnical evaluation. It is interesting to note the similar results for Caucasian and Asian ethnicities, despite the differences in MAF of T allele into these distinct populations.

Although, to the best our knowledge, this is the first meta-analysis to focus in to determinate the association between a polymorphism in IL2 *gene* and periodontitis and brought significant number of participants, the meta-analysis showed important limitations that should be noted.

First, five studies were included in quantitative syntheses. This limited number of included studies is not sufficient to show robustness in results and may be a source of bias. However, seen this limitation we have performed accurate statistical methods to validate our findings. Second, important factors associated with patients were not available in included articles. A complete evaluation about adjusting factors such as: gender, smokers and non-smokers, stratified age data and others conditions that influence the development of periodontitis was not possible due to the limited included data in the studies. Third, periodontitis is a clinical condition that receives several classifications. An evaluation take in base others types of periodontitis also was not possible. Besides, a new classification for the disease has been proposed and must be considered by future studies [46]. Fourth, almost all calculations were interfered by significant heterogeneity and use of Random-effects statistical model. Heterogeneity is a statistical tool to prove how the studies are inconsistent. It may be an important fact in meta-analysis calculations because the presence or absence of true heterogeneity affects the statistical model applied on the included data [47]. The use of Random-effects in the results from meta-analysis promotes more weight to studies containing a small sample-size, what may not be considered totally trustworthy [48]. Fifth, several non-significant associations were found in the meta-analysis calculations. However, the non-significant *P* value does not always reflect the absence of clinical relevance [49] what led us to consider these results with caution.

Furthers studies must be focused in attempt the real association between − 330 T/G polymorphism and periodontitis correlated with others factors that may promote progression of the disease (alcohol consumption, smokers patients and others clinical classifications for periodontitis) as well as others ethnical groups. Likewise, future studies to determinate the influence of haplotypes between this polymorphism with others genetic variations are required with more knowledge about genetic factors and risk of periodontitis development.

## Conclusions

In conclusion, into the limitations, this current meta-analysis showed a non-significant association between the − 330 T/G polymorphism in IL2 *gene* and CP in any allelic evaluation with increased heterogeneity and absence of publication bias.

## Supplementary information

**Additional file 1.** PRISMA 2009 Checklist.

## Data Availability

Not applicable.

## Abbreviations

AgP: Aggressive Periodontitis
CI: Confidence Intervals
CP: Chronic Periodontitis
I^2^: Heterogeneity
IL: Interleukin
MeSH: Medical Subjects Headings
OR: Odds Ratio
PRISMA: Preferred Reporting Items for Systematic Reviews and Meta-analyses
